# Democracies Restricting Democratic Rights: Some Classical Sources and Implications for Ethics of Biometrics

**DOI:** 10.1100/tsw.2011.47

**Published:** 2011-03-01

**Authors:** Frank J. Leavitt

**Affiliations:** Medical Ethics Centre, Faculty of Health Sciences, Ben Gurion University of the Negev, Beer Sheva, Israel

**Keywords:** Athens, biometrics, data sharing, democracy, ethics, ideology, Locke, Milton, Reformation, revolution, rights, security, Socrates

## Abstract

Ancient Greek and 17 century English philosophy are not usually discussed along with the ethics of biometrics and data sharing. Academic ethics today, however, suffers from a lack of background in classical texts. We may discuss whether biometrics and data sharing are consistent with democracy, but if we do not know what democracy is, then we cannot know what actions are consistent with it. I shall discuss how and why democracies have restricted the rights of their citizens. I will give the most attention to two paradigms that have most influenced modern democratic thinking: 17 century English democracy and ancient Athens. I do not accept the dogma that the Athenians were obviously wrong to try and then to condemn Socrates. His death-loving doctrine could not but have weakened the will of the youth to work and fight for the good of Athens. I will try to understand the Athenians' point of view and their need to defend their security. At the end, I will apply these lessons to biometrics and data sharing for security reasons.

## INTRODUCTION

It may be surprising to see ancient Greek and 17th century English philosophy among discussions of the ethics of advanced methods of keeping track of what people are doing in such contexts as biometrics, data sharing, and border control. I believe, however, that the academic field of ethics today suffers from a lack of background in the classical texts. A major ethical question about biometrics and data sharing for security purposes has to do with keeping these methods consistent with ideals of democracy. However, this requires familiarity with history and the philosophy of democracy. If we do not know what democracy is or is not, then we cannot know what actions are consistent with democracy and what are not. I shall discuss how and why democracies have restricted the rights of their residents and citizens. I will use several examples from the U.S., but will give the most attention to 17th century English Reformation democracy and to democratic Athens and the trial of Socrates. I do not accept the dogma that the Athenians were obviously wrong to try and then to condemn Socrates. I will try to understand their point of view and their need to defend the security of Athens. At the end, I will try to apply what I learned to biometrics and data sharing for security reasons.

"Democracy", as everyone knows, means rule by the people. I would call this a central ideal of democracy. However, that does not tell us what people are going to rule and what is to be the method of ruling.

I see no better way to understand what *rule by the people* means in democracy than to examine classic texts in the philosophy of democracy and classic examples of democratic societies. I do not believe that there is any absolute, Platonic idea or concept of a democracy by which purported democracies can be measured. To lay a concept down *a priori* is only to try to say that I know better than anybody else. Instead of such presumptuousness, I prefer to try to find out what democracy is by examining societies and philosophies that are commonly acknowledged as democratic. Democracy is as democracy does.

The method by which the people rule can be anything from a formal constitutional democracy with divisions of powers, checks, and balances, etc., to an informal New England town meeting, to rule by an unchecked and violent mob. However, whatever the method of ruling, a rule by the people of a certain country, municipality, or area is never a rule by all of the people resident in that country, municipality, or area. It is always a rule by those people who happen, by virtue of age, gender, merit, inheritance, revolution, conquest, political maneuvering, skillful propaganda, seizure, etc., to be the ruling people.

It is obvious that democracy is not necessarily rule by all residents. No democracy has ever allowed small children, the severely retarded, or the severely mentally ill to participate in the ruling process. Of course, there is the children's liberation movement, which would like to allow children all the rights that adults have, and there is the antipsychiatry movement that would like to allow the same for the mentally ill, if indeed the movement accepts the concept of mental illness at all. However, there is no way in which an infant can exercise the right to participate in democratic rule. The same can be said about the severely retarded and the severely mentally ill. Even when democratic rule is violent mob rule, not everyone has the ability to get out, join the mob, and take part in the violence.

In bioethics, there is a concept of surrogate decision making. Children and the mentally incompetent are thought of as having the right to autonomous decision making because they have a surrogate, a parent or perhaps a court-appointed guardian, to decide for them. However, this is a sort of legal fiction and not a very good fiction at that. To say that someone is exercising the right to autonomous decision making because someone else is deciding for him is an oxymoron. The concept of autonomy simply does not apply to people who cannot decide for themselves with a certain degree of understanding of what they are deciding.

Democratic states, as we shall see, have denied full democratic rights, including the right to participate in political decision making, to mentally competent adults. So have philosophers of democracy. This may cause them to deserve criticism as states, as other political bodies, or as philosophers. But in my humble opinion, this does not make them any the less democratic.

Many examples can be brought of restrictive, but democratic, states and restrictive, but democratic, philosophies of democracy. Here are some: restrictions on religious freedom in England under Cromwell, as Cromwell's philosopher, John Milton, urged in his political essays[1]; restrictions on who is to receive democratic rights in a commonwealth in John Locke's *Letter on Toleration*; slavery in the pre–Civil War U.S.; restrictions on Native Americans in the U.S. and Canada[2,3]; continued segregation of Black Americans, far into the 20th century, supported by the Democratic Party in the south; the forcible internment, by the U.S., of Japanese, including American citizens of Japanese descent during World War II[4]; and, of course, the trial and execution of Socrates in democratic Athens[5].

It is a mistake to use the word "democracy" as an evaluative term. People often intend the word "democratic" to bear the implication that a thing is good. People often intend the term "nondemocratic" to bear the implication that a thing is bad. But these are mistakes. The concept of democracy has got nothing to do with good or bad. It has to do with who governs and how. A democratic regime can be an evil regime.

There is a dogma, which may even be dangerous to challenge, among academics, especially among philosophers. According to this dogma, the acts of the Athenians in bringing Socrates to trial and then sentencing him to death are eternal paradigms of all that is evil: the closed society, fascism, the denial of academic freedom, the boorish hatred of free enquiry and academic freedom, etc. This paper, however, will challenge that dogma and argue that the Athenians had no choice. They had to protect their democracy and put a stop to words and deeds that were weakening the youth. While not denying I.F. Stone's political interpretation of the charges against Socrates[6], I emphasize another aspect: the otherworldly, almost cult-like, death-oriented nature of his teachings.

## REVOLUTIONARY DEMOCRACY IN 17TH CENTURY ENGLAND

The democracy of the Protestant Reformation in 17th century England is not only interesting in itself. It is important because it was there that philosophies were developed that formed the basis for democratic thinking in the English-speaking world until this day.

An overly brief theological history will be followed by some remarks about how the philosophers of this democracy saw the need to restrict democratic freedoms in order to preserve the security of the democracy itself.

England had two revolutions in the 17th century. The first was the bloody revolution led by Cromwell, who beheaded King Charles I on January 30, 1649[7]. The second, not quite peaceful, but much less bloody, was the Glorious Revolution of 1688[8].

Cromwell's revolution, the political side of the religious Reformation, was a major milestone in the development of democracy in Western Europe. The Protestant Reformation was a movement against the centralization of religious authority in the Church and a movement for the devolution of religious authority into the hands of the people, *the priesthood of all believers*, according to one of the slogans of the time. The Bible, which had existed only in Hebrew, Greek, and Latin, was translated into English and European languages, probably with the aid of Jews who had fled forced conversion and slaughter during the *Almohade* (*Al Muwahadun*) rule of Spain (1130–1269) and who were finally driven out of Spain by the Christians in 1492. The new ability to read the Bible led to a demand for the right of individuals to interpret it for themselves.

I wish to suggest that this reversal of the hierarchy, which was central to and perhaps the main point of the Protestant Reformation, was parallel to and likely a conscious or unconscious imitation of a process that went on in Judaism from the time of the destruction, by the Roman conquerors, of the Second Temple. With no more Temple, our *Cohenim* or priests, a hereditary status in Judaism, no longer had any more function than making a few blessings in some of our prayers. Our rabbis, religious scholars, had served during the time of the Temple as judges in courts of law that had full authority to decide cases ranging from infringements of religious ceremonial law (like Sabbath observance) to monetary disputes to capital crimes[9]. But approximately around the time of the destruction of the Temple, rabbinical ordination, *smicha*, ceased. Since then, rabbis have had no more power than what the people have been willing to give them. Two disputants can agree to argue their case in front of a rabbi or rabbis and to accept their decision. But the rabbinical power rests only on the consent of the people. They do have legal authority in matters of Jewish marriage and burial, as have Christian and Islamic religious leaders for their own people. But this authority was instituted by democratically elected governments. Religious authority no longer comes from the top down, but from the bottom up. This is exactly the central idea of the Protestant Reformation[10]. A Christian congregation is run by elders who derive their power from the consent of the people.

As we shall see, such major philosophers of the Protestant Reformation opposed equal rights for all people. This also has a precedent in Judaism. Judaism is not a racist religion. People of all races can convert to Judaism and their children are just like any other Jew. But the convert himself is under various restrictions. Jewish Law opposes giving him any position of political authority[11]. This is all the more so for a non-Jew who has not converted to Judaism.

It was a short step from ecclesiastical democracy to civil democracy. Just as no church had the authority to dictate spiritual matters to the people, so no king or governmental hierarchy had the right to dictate civil matters to the people. A democratic rule by the people through an elected parliament was, at least theoretically, put into effect by Cromwell's revolution and the beheading of King Charles I in 1649[7].

Cromwell's philosopher was the poet John Milton, who wrote political-theological-philosophical essays to defend the beheading of King Charles I and to promote the new Commonwealth. Milton did not hesitate to start placing restrictions on democratic rights. Total freedom of religion was to be granted to all except Roman Catholics[10].

In thinking about the pros and cons of Milton's, and as we shall see later, Locke's, denial of religious rights to Catholics, one already sees the concepts that must be discussed with respect to the ethics of restricting democratic rights in order to preserve the security of democracies today. On the one hand, one can easily see the reason in such a restrictive position. The Catholic Church thinks of itself as *the one true catholic and apostolic church.* But the word "catholic" means universal. This means that it is supposed to be for everyone with no exceptions. So whoever professes the Catholic faith may be expected to make a serious effort to get others to profess the same faith. But anyone whose faith requires that everyone else in the world adopt the same faith is dangerous to everyone whose conscience has led him to different religious or nonreligious convictions. So, with all due respect to Catholics, one can understand the Reformation idea that Catholics must be disallowed in the new Commonwealth. Here is a restriction of democracy to protect democracy, which by extension might be applied to justify allowing democratic governments much leeway in security checks, biometrics, and even interrogations, in order to protect democracy.

On the other hand, and with all due respect to Protestants, there is a certain spiritual depth to Catholicism that was thrown out with the Reformation: the recognition that, if I may borrow a phrase from Shakespeare, there is more in heaven and earth than is dreamed of in any purely rational philosophy; the way in which Church architecture and music inspire pious feeling; an understanding of, or at least a deep interest in, the metaphysical side of existence, the soul, the nature of the afterlife, angels, demons, and God. The Catholic universities, moreover, carried on the classical philosophical tradition. Although modern philosophy and science revolted against this tradition, their revolt would not have been possible without concepts that they inherited from this tradition. I doubt that Milton himself could have written *Paradise Lost*[12] without concepts like the Trinity, angels, devils, etc. that Catholic tradition preserved throughout the centuries.

It is not only in science, but also in religion that even revolutionaries, if I may borrow a phrase attributed to Newton, stand on the shoulders of giants.

Ridding the Commonwealth of Catholicism might have rid it of much that is beautiful and sublime in human life. If Catholicism had simply sought a religious conquest of the world, while contributing nothing to human culture, I might have agreed with Milton's and Locke's desire to suppress it. Because of its great contribution to human culture, however, it should have been encouraged to flourish, while we remained vigilant against all religious conquest at the same time. It should be noted, moreover, that since the second half of the 20$^{th}$ century, Catholicism has become quite tolerant of other faiths.

Similarly, in our own day, the unchecked use of security measures might be an efficient way to preserve democracy. But at the same time, it might castrate the very democracy we want to preserve by emptying life of its most sublime experiences. Here I only echo similar fears of the highly technological society that were voiced in Orwell's *1984*[13], which was first published in 1949; Huxley's *Brave New World*[14], first published in 1932; and Russell's *The Scientific Outlook*, which predated them both in 1931. Some of these fears may seem naïve today. For example, Russell wrote: "By means of governmental microphones the censors will listen in to … conversations, and if these should at any time become tinged with sentiment disciplinary measures will be adopted[15]." Russell may have exaggerated, but one wonders what psychologically repressive effect there will be on our emotions if we are increasingly observed, measured, photographed, and recorded.

John Locke is better known than John Milton as a philosopher of 17$^{th}$ century English Reformation democracy. Cromwell's Commonwealth died when Cromwell himself died. There was no one with his leadership abilities. Parliament had no choice but to restore the monarchy by calling Charles II to take the throne. Under the restored monarchy, Roman Catholicism increased in influence. The Protestants, in turn, invited William of Orange to conquer England for their cause. William's army, which invaded England in 1688, was unopposed. The philosopher of the new Protestant rule was John Locke. Although much of Locke's philosophy of democracy was foreshadowed by Milton, it is Locke who deserves to be called the Father of Modern Democracy. He influenced the American and the French Revolutions through their philosophers. His words form part of the U.S. Declaration of Independence. Locke's political philosophy was based on the idea of the social contract, which symbolized a situation in which a ruler rules only in virtue of having been appointed by the people[16]. The ruler is more or less an employee of the people and has no powers other than those that the people give him. The words of the U.S. Declaration of Independence, i.e., "governments are instituted among men, deriving their just powers from the consent of the governed," sum up much of Locke's philosophy. In the social contract as Locke saw it, the people instituted a commonwealth with a government or ruler, what Locke called a *magistrate,* to protect the people's lives, liberty, and property.

The government had no further powers, certainly no power to interfere in the religious faith of the people. Locke wrote in his *Letter Concerning Toleration* (I quote from William Popple's 1689 translation from Locke's Latin original): "… [T]he care of souls is not committed to the civil magistrate, any more than to other men….it appears not that God has ever given any such authority to one man over another as to compel anyone to his religion…. no man can, if he would, conform his faith to the dictates of another. All the life and power of true religion consist in the inward and full persuasion of the mind; and faith is not faith without believing[17]."

Even if we wanted to turn over to a government the power to decide our faith for us, we would not be able to do so. For faith is a matter of inward conviction. We can accept a government's dictates as to our faith only if we are convinced of what they say. But if we are really convinced, then the dictate is not a dictate, but a matter of our own free choice.

It may come as a surprise, however, to learn that although Locke was not willing to allow a commonwealth to dictate what someone's religious opinion ought to be, he was willing to dictate what it ought not to be. Locke did not want to allow Mohammedans into his commonwealth because they were loyal to foreign religious authority. As he says: "It is ridiculous for anyone to profess himself to be a Mahometan only in his religion, whilst at the same time he acknowledges himself bound to yield blind obedience to the Mufti of Constantinople…[etc][18]." Similarly, he wrote elsewhere of Catholics: "Papists are not to enjoy the benefit of toleration because where they have power they thinke them selves bound to deny it to others. For it is unreasonable that any should have a free liberty of their religion, who doe not acknowledg it as a principle of theirs that noe body ought to persecute or molest another because he dissents from him in religion[19]." Locke wrote four different works on toleration. The reasons why he sometimes wrote against Roman Catholics and sometimes against Muslims are a subject for Locke scholars[20].

Nor was he was willing to allow atheists. As he says: "Lastly, those are not at all to be tolerated who deny the being of a God. Promises, covenants, and oaths, which are the bonds of human society, can have no hold upon an atheist[18]."

Both of these restrictions are for the sake of security of person or property. Locke's reasoning is that someone who is willing to allow a foreign religious or other authority to dictate his behavior cannot be counted on not to betray his country in time of war or other national danger. Someone who is not bound by his promises, covenants, and oaths cannot be counted upon to behave honestly in business, defense, or other matters.

Of course, it can be questioned whether atheists are so lacking in trustworthiness. The reasoning behind Locke's opinion seems quite clear. He did not believe that anything short of belief in God could give us a reason to be honest. I can *almost* accept Locke's view because I have never been convinced that any nonreligious philosophy has given adequate reasons why I should be honest and not lie and deceive. Let us take one example, utilitarianism. If I am convinced that I can give more happiness and prevent more suffering for more people if I am a deceitful person, then how can utilitarian philosophy forbid me to be deceitful? If, on the other hand, I am convinced that God has commanded me to be trustworthy and if I believe, moreover, that living a meaningful life requires me to adapt my will to God's, then I should be less likely to lie.

The matter, however, is not so simple. It is hard to find a religion free of scandals among its most pious practitioners. It is, on the other hand, not hard to find nonbelievers who are very honest and trustworthy.

Locke also wanted to root out "adultery, fornication, uncleanliness, lasciviousness, idolatry, and such-like things[18]." But because such like things do not seem to bear directly on security, I shall not discuss them here. Religious, philosophical, and ideological beliefs that can lead to behavior dangerous to the survival of a democracy do bear on security and must be discussed here. This, I think, is the relevance of Locke's writings on toleration to the topic of the ethics of biometrics.

Should tests designed to identify dangerous religious, philosophical, and ideological beliefs be used at airport security checks, in decisions whether to grant someone a visa or citizenship, in job interviews, or even in population-wide screening of citizens and alien residents? Should e-mails be screened for evidence of such beliefs? I cannot take a dogmatic position on this question. A society has the right to defend itself. On the other hand, too much digging into personal matters can make the society not worth living in. I am not yet sure where to draw the line.

## THE TRIAL AND EXECUTION OF SOCRATES

Everyone knows the story. Socrates was a good citizen of Athens and had fought bravely in the Persian war, but his way of life was idiosyncratic. He was a teacher, but rather than founding a school as did his student, Plato, and Plato's student, Aristotle, Socrates taught for free in the marketplace and taught everyone who was willing to be taught. His method of teaching was to ask questions. Rather than conveying information to the student, Socrates would interrogate him, helping the student to discover truths by himself. Socrates, however, at least as we learn about him through Plato's *Dialogues*, did not really believe that he was helping the student to discover truths. He really believed that the student already knew the truth and that what appeared to be discovery or learning was really uncovering and revealing truths that already lay deep within the student's soul. According to this doctrine, called the doctrine of *anamnesis*, we are born knowing all the truth. However, when we are born, we forget that we know it. Life is a process of recalling what we knew before birth. Plato's dialogue, the *Meno*, shows Socrates demonstrating his doctrine of *anamnesis* by questioning an illiterate slave boy and showing that the slave boy knew Pythagoras's theorem about right triangles all along[21]. Whether Socrates really proves that the slave boy knew the theorem or simply leads him along is a matter for debate.

Socrates was part of the wonder that was ancient Greece: free and open discussion, philosophy, theater, sport, science, mathematics. Athenian society seems best to have exemplified these values. It was not exactly a free society. Slaves, women, children, those who owned no property, and foreigners were denied political rights, but Athens was a democracy in the strict sense of the word: a society ruled by the people; ruled, that is to say, by members of the ruling people.

Things were not perfect in Athens in Socrates' time. There was a school of commercial teachers, the Sophists, operating. Teaching for money was contrary to Socrates' values. They taught rhetoric and debate and have been accused of teaching people how to make the better thing look worse and the worse thing look better. Their questioning seems to have led to doubts about traditional Athenian religion and values.

No society is perfect and Athens seems to have been a pretty good society as far as societies go.

Two tragic events, however, shook Athenian society to its foundations. First was the war between Athens and Sparta, which began in 431, according to the Christian calendar, and ended with Athens' crushing and humiliating defeat in 404. The second was a great plague that, from 430 to 426, killed about a third of the Athenian population[22].

Incidentally, the historical facts and dates that I cite are easily found in the literature. But I take some of them from M.I. Finley's masterful essay "Socrates and Athens"[22], which lays them out for us on a platter. As we shall see, however, I cannot accept Finley's view of the reasons why Socrates was brought to trial.

Socrates was summoned before the 501 men of the town assembly of Athens to be tried on three counts: denying the gods of the city, introducing new gods, and corrupting the youth. He defends himself at length, arguing that he has only done good, never harm, by teaching young Athenians, and that he has never denied the official gods of Athens. As for the charge that he corrupted the youth, he points out that many of those sitting in the assembly had been his students when they were young. He challenges his accuser to identify one of them whom he corrupted. The challenge was not taken up. Yet, he was convicted by a majority of 361 to 140 and sentenced to death[22].

The execution would normally have taken place very soon after the verdict, but there was a custom that the city was not to be polluted by executions during the time of the annual pilgrimage to Delos. The ceremony marking the beginning of the pilgrimage took place the day before the trial and the execution could not take place until after the pilgrimage ship returned. This allowed a good, long period of time and an excuse for Plato to write dialogues depicting Socrates as carrying out philosophical conversations with his students while they visited him in prison. Plato's dialogues covering the time from shortly before the trial until Socrates' death, the *Euthyphro*, the *Apology*, the *Crito*[5], and the *Phaedo*[23], should be part of any education. Socrates did not see his execution as necessarily a bad thing, so sure was he of reincarnation and of a future world of light and truth in the place of our present world of shadow and falseness. He calmly drank the cup of poison hemlock as he consoled his friends.

Socrates became so much a hero of philosophy that when I first heard a lecture about his fate in an undergraduate philosophy course, the professor broke down in tears as he described Socrates' death. Philosophers and classical scholars discuss and debate the exact reasons why the Athenians tried him and put him to death. But it is usually taken as absolute and eternal truth that they were wrong to do so. Finley says that this trial "demonstrates that at the time the people of Athens had retreated in panic from their high ideals." He suggests that they behaved "irresponsibly and viciously," that they looked "after their private interests first." He does allow that they were not "incurable" and that after this trial "legal persecution for ideas [came] to an abrupt end[22]." However, he does not try to explain exactly why such persecution came to this abrupt end.

Finley's account is also unsatisfying with respect to the specific accusations. Socrates was accused of impiously denying Athens' accepted gods and introducing new ones. Finley argues with good reason that these accusations were unfounded. There was in Athens in this generation, Finley says, an "upsurge of magic and superstition" and "swarms of private soothsayers, diviners and oracle mongers." Why then should Socrates have been singled out? Finley points out, moreover, that Socrates was "a man of very deep piety" and "scrupulously performed the sacrifices and other rites[22]." Finley makes it seem as if there was no base at all for the accusation of impiety. But if there was no base for it, shouldn't this have been obvious to his accusers? Also, if there was no base for the accusation, why did the jury convict him?

I would like to make an alternative suggestion. According to what we know of Socrates' philosophy through Plato's *Dialogues*, Socrates believed in metaphysical "forms", the realities of which this world is only a poor and confused imitation. The main thing that we have to do in life is to investigate these forms, to try to understand them better. This was the purpose of the method of teaching that Socrates practiced on Athens' youth, to encourage people, perhaps especially the youth, to look inside themselves, make contact with these metaphysical forms, and devote themselves to studying them rather than dealing with the daily affairs of an imperfect and confused world. His teaching was a kind of religious indoctrination. He was like a missionary for the theology of forms. I suggest that this preoccupation with metaphysical forms was the basis for all three of the accusations.

By "not believing in the gods in which the city believes," I suggest is not meant to assert that they did not exist or to fail to perform the prescribed rituals in their honor. What is meant is lowering their status. Socrates had no reason to deny that they existed. He had no problem going through the accepted rituals. He may even have done so with enthusiasm. But the accepted gods were nonetheless second-class entities. His metaphysical forms were first class. They occupied his attention during most of his waking hours and he encouraged his pupils to follow him in this. The primary status of the metaphysical forms was so dear to Socrates that, as he makes very clear in his deathbed dissertation in the Phaedo, death – where we may meet these forms in their purity face to face – is preferable to living in this second-class confused and vague world[23].

The metaphysical forms are the new gods that Socrates introduced. The forms were in every way Socrates' chief gods. The medieval Catholic philosopher, Thomas Aquinas, compared the angels to the Socratic-Platonic metaphysical forms[24]. But what are angels if not a kind of deity?

This doctrine has its implications with respect to the plague that had killed a third of the Athenians, with respect to the Peloponnesian war that Athens had lost, and with respect to the accusation of the corruption of the youth.

As for both the plague and the war, how can the gods have been expected to protect the Athenians if the latter allocate them a second-class status? And here Socrates not only preferred his metaphysical forms to the municipal gods, but had the audacity to teach such preference to the youth. He clearly presented a danger to the security of Athens.

With respect to the corruption of the youth, the matter is even more serious. He corrupted them in the first place by encouraging them to prefer his metaphysical forms to the municipal gods. Of course, this crime will not be taken very seriously by moderns who do not believe that God or gods exist anyway, and so see no danger in taking them lightly. But there is another danger here that even the most antimetaphysical materialist ought to recognize. In encouraging the youth to turn from worldly pursuits to the philosophical analysis of the metaphysical forms, he would have been acting against their enthusiasm to restore the glory of the city of Athens and fight to defend it.

To get a feeling for what it meant to be a conscript soldier in ancient Greece, I highly recommend V.D. Hanson's book on the life of the ordinary Greek infantryman, *The Western Way of War: Infantry Battle in Classical Greece*[25]. You had to fight in the phalanx, a tight formation in which you would carry a very heavy wooden shield in addition to your spear. You also wore a heavy helmet and armor. Your eyes were so filled with sweat that they stung and you could barely see. With the noise of the clashing shields and spears, the pipers conveying orders, the screams of wounded and dying men, and the bloody confusion, it must have been a nightmare for any but the most calm and dedicated soldier. Any armor will have its soft spots at the joints of the body, to allow the soldier to move. So a favorite tactic for bypassing the shield and penetrating the armor was to stab upward from below. The pain and the humiliation of such a death challenges the imagination. Yet patriotic men continued to volunteer into old age, long after their compulsory service was finished.

What kind of soldiers would young people be, however, if they believed that the contemplation of the metaphysical forms was more important than anything that takes place in this lower world? How important could the defense of Athens be to them anyway? What did it matter whether Athens was free or under the hand of a Spartan garrison if the political situation did not interfere too much with the contemplation of the metaphysical?

This was not a harmless corruption of the youth. It was a corruption of the sort that endangered the security of a state that depended on an able-bodied and patriotic youth for its defense. How can we count on youth who believe that to be a philosopher is to pursue death and dying[23]? We have to start re-examining our philosophical dogmas about Socrates' absolute righteousness and the Athenians absolute depravity. I am not sure I would not have brought charges against him myself. I would, however, have asked for less than the death penalty because I believe that Socrates' intentions, if not his deeds, were good.

As already mentioned, as bad as Finley thought the Athenians to be, he allows that they were not "incurable". He states that after Socrates' trial, "legal persecution for ideas [came] to an abrupt end[22]." But he fails to explain why this persecution came to an end. I submit that it came to an end because there was nobody left to persecute, nobody, that is, who was willing to risk receiving Socrates' fate by going around corrupting the youth. I submit that Socrates' trial was a show trial and a warning for others. Its deterrence effect is clear from the story about Aristotle who, when he himself was in danger of prosecution, fled Athens saying he would not give the Athenians another chance to sin against philosophy. A.H. Chroust has discussed the background of the charges against Aristotle in his association with the Macedonian conquerors[26]. His teaching of science and philosophy drew no such opposition.

The purpose of Socrates' trial was not to put an end to free enquiry, but to put an end to indoctrination that endangered the security of the state. The subsequent behavior of the Athenians in not preventing Plato and Aristotle from establishing and running their schools helps makes the message clear: go ahead and enquire freely, study mathematics and physics, art and literature, enquire about ethics, the best ways of life for a human being, and about politics, the best form of organization and rule for the state. But just do not go around confusing young people's minds, making a mess of their enthusiasm for this world and of their enthusiasm for trying to make it a better world, destroying their morale and their will to fight as soldiers in the defense of their land.

Again we have seen how a democracy had to restrict freedom in order to defend its existence. Milton and Locke were afraid of loyalty to foreign authorities. The Athenians were afraid of threats to the will of the youth to fight and work for the good of Athens.

## APPLICATION TO BIOMETRICS AND TO DATA SHARING

One theme has repeatedly appeared in our brief examination of democracy in ancient Athens and in Reformation England: the response of democratic societies to religious, philosophical, or ideological doctrines that may endanger the security of the society. We have seen that the response can be the restriction of democratic rights or even the death sentence.

Biometrics is usually thought of as dealing with physical data, fingerprints, voice, eyes, anatomy of the hand, posture, etc. Technologically, it ought to be quite feasible to add religious, philosophical, and ideological data to one's biometric profile, making possible a measure (metric) of the mental aspect of one's life (bio). Perhaps this would be an extension of the word "biometrics", but isn't extending meanings part of how languages develop? I have quite a number of publications available online that can be computer scanned along with my e-mails, telephone, and Skype conversations. I should not be surprised if the technology already exists to enable computers to work these data up, automatically, into my religious, philosophical, and ideological profile. It should then be possible to work out automatically the statistical likelihood of my teaching doctrines that will weaken the resolve of young people to work to strengthen and to fight to defend their countries, or doctrines, which would incite rebellion or encourage the enemy. If this likelihood should pass a certain threshold, then red lights will automatically go on whenever my name comes up in police stations, border crossings, and the hiring offices of employers. The data can, moreover, be shared with friendly countries. I shall not be surprised if experts in this field tell me that I am boring them because this sort of thing is not only possible, but has been in use for years, not only in cruel dictatorships, but in the nicest and most democratic societies.

A biometric profile including religious, philosophical, and ideological data might be looked at as taking to an extreme the trend to use mathematical science for prediction and control. Here we would use it to predict and control the behavior of immigrants, travellers, and perhaps, eventually, everyone. I think that authors like Capra[27], for example, have made too much of prediction and control as a supposedly unique feature of the modern use of science. There is nothing modern about it. In ancient India, China, and Greece, medicine was used to identify predictors of health and disease, and to control diets and lifestyles to try to maintain health. But although medicine, in its ideals if not always in its practice, is benign, biometric religious, philosophical, and ideological profiles may seem to be diabolical.

Are religious, philosophical, and ideological biometric profiles, whether they exist yet or not, really so diabolical? Personally, I do not mind the invasion of privacy. Not much privacy exists any more anyway. What does trouble me is the threat to academic freedom and other forms of free intellectual enquiry. A democratic society has a right to defend itself, but I do not know if a society that forbids free intellectual enquiry is a society worth defending. We must, however, decide what true academic freedom, genuine intellectual enquiry, is, and what it is not. But is the act of intentionally weakening one's country and encouraging one's country's enemies an exercise of academic freedom? Even if it is, this would suggest at most that a university has no authority to interfere with what takes place in the classroom or with what ideas a lecturer publishes. The police and other law enforcement agencies are another matter. After the American poet Ezra Pound broadcast profascist speeches to American and British troops during World War II, clearly aimed at weakening their will to fight, the Americans had no hesitation about arresting him at the end of the war.

Let us recall that the Athenians did not bring Socrates to trial for philosophical enquiry. They tried him for corrupting the youth. And they did not interfere with Plato's Academy, where every philosophical, scientific, religious, and literary idea could be freely discussed. They tried, moreover, to bring charges against Aristotle, not because of his philosophical teaching, but because, as the teacher of Alexander, he had been allied with the Macedonian conquest.

There is clearly a distinction between free enquiry and sedition. The precise criteria for this distinction, however, have not yet been defined in detail.

## ACKNOWLEDGMENT

The author wishes to thank Barbara Prainsack for her helpful comments and for her never-ending support and encouragement.
